# Leveraging Large-Scale Electronic Health Records and Interpretable Machine Learning for Clinical Decision Making at the Emergency Department: Protocol for System Development and Validation

**DOI:** 10.2196/34201

**Published:** 2022-03-25

**Authors:** Nan Liu, Feng Xie, Fahad Javaid Siddiqui, Andrew Fu Wah Ho, Bibhas Chakraborty, Gayathri Devi Nadarajan, Kenneth Boon Kiat Tan, Marcus Eng Hock Ong

**Affiliations:** 1 Programme in Health Services and Systems Research Duke-NUS Medical School Singapore Singapore; 2 Institute of Data Science National University of Singapore Singapore Singapore; 3 SingHealth AI Health Program Singapore Health Services Singapore Singapore; 4 Health Service Research Centre Singapore Health Services Singapore Singapore; 5 Department of Emergency Medicine Singapore General Hospital Singapore Singapore; 6 Department of Statistics and Data Science National University of Singapore Singapore Singapore; 7 Department of Biostatistics and Bioinformatics Duke University Durham, NC United States

**Keywords:** electronic health records, machine learning, clinical decision making, emergency department

## Abstract

**Background:**

There is a growing demand globally for emergency department (ED) services. An increase in ED visits has resulted in overcrowding and longer waiting times. The triage process plays a crucial role in assessing and stratifying patients’ risks and ensuring that the critically ill promptly receive appropriate priority and emergency treatment. A substantial amount of research has been conducted on the use of machine learning tools to construct triage and risk prediction models; however, the black box nature of these models has limited their clinical application and interpretation.

**Objective:**

In this study, we plan to develop an innovative, dynamic, and interpretable System for Emergency Risk Triage (SERT) for risk stratification in the ED by leveraging large-scale electronic health records (EHRs) and machine learning.

**Methods:**

To achieve this objective, we will conduct a retrospective, single-center study based on a large, longitudinal data set obtained from the EHRs of the largest tertiary hospital in Singapore. Study outcomes include adverse events experienced by patients, such as the need for an intensive care unit and inpatient death. With preidentified candidate variables drawn from expert opinions and relevant literature, we will apply an interpretable machine learning–based AutoScore to develop 3 SERT scores. These 3 scores can be used at different times in the ED, that is, on arrival, during ED stay, and at admission. Furthermore, we will compare our novel SERT scores with established clinical scores and previously described black box machine learning models as baselines. Receiver operating characteristic analysis will be conducted on the testing cohorts for performance evaluation.

**Results:**

The study is currently being conducted. The extracted data indicate approximately 1.8 million ED visits by over 810,000 unique patients. Modelling results are expected to be published in 2022.

**Conclusions:**

The SERT scoring system proposed in this study will be unique and innovative because of its dynamic nature and modelling transparency. If successfully validated, our proposed solution will establish a standard for data processing and modelling by taking advantage of large-scale EHRs and interpretable machine learning tools.

**International Registered Report Identifier (IRRID):**

DERR1-10.2196/34201

## Introduction

### Background

Across the globe, there is increasing demand for emergency department (ED) services [1,2]. Increased ED visits have resulted in overcrowding and long waiting times [3-5]. Furthermore, adverse patient outcomes have been reported, such as mortality [6], poor patient satisfaction, and high costs [7,8]. As the first layer of emergency care in an ED, triage plays an essential role in assessing and stratifying patients’ risks and ensuring that the critically ill receive appropriate emergency treatment promptly [9].

The triage process is commonly conducted by medical staff based on their own clinical experience, the patients’ symptoms, and basic information obtained from patients during their presentation to the ED. To make this critical step more objective, triage systems have been introduced. Some examples of triage systems include the 5-level Emergency Severity Index [10] in the United States, the Australasian Triage Scale [11] in Australia, and the Patient Acuity Category Scale (PACS) [12] in Singapore. They are simple and easy to use but subjective and static. These scores are based on symptoms, but many critically ill patients may not have apparent symptoms when they arrive at the ED and their conditions deteriorate rapidly during their stay in the hospital. To address this limitation, more dynamic and accurate risk prediction tools are required for better patient monitoring throughout the ED journey [13].

In response to this gap of needs, researchers are interested in developing multivariable predictive models and clinical scores to identify patients in the ED at risk of adverse outcomes such as admission [14,15], death [16], cardiac arrests [17], and intensive care unit (ICU) admission [18]. Models such as these are primarily based on patient information, vital sign instability, changes in laboratory results, and administrative records. However, some parameters may appear similar between high-risk patients and other patients during an ED visit, making the prediction models less accurate.

Additional risk factors such as comorbidities, underlying chronic diseases, past hospitalization history, and other patient-related factors should be considered [19]. Furthermore, nonpatient factors are also integral components of patient care that can impact patient outcomes. Research has identified emergency boarding as a risk factor for mortality [6]. In addition, mortality rates were found to be higher for patients admitted during periods of high ED crowding regardless of their demographic characteristics, comorbidities, or primary diagnosis [20]. Changes in shift and high patient-to-nurse ratios have also been factors of concern [21].

In building predictive models, both traditional statistical methods and machine learning tools have been thoroughly investigated. Logistic regression is the most commonly used tool to construct multivariable prediction models [16,22,23]. In recent years, machine learning and artificial intelligence (AI) have gained popularity as tools for improving model performance. Fernandos et al [24] conducted an in-depth review of the current state of AI-based clinical decision support systems for triage. A recent study in the United States demonstrated the value of machine learning models for admission prediction in near real time [13].

While AI has proven successful in developing triage and prediction models, its solutions are often black box models, limiting model interpretation [25] and clinical adoption [26]. Consequently, efforts have been made to develop sparse predictive models by leveraging machine learning and conventional statistical analysis. Ustun and Rudin [27,28] proposed Supersparse Linear Integer Model–based methods for developing interpretable scoring systems. Xie et al [29] developed the interpretable machine learning–based AutoScore framework and used it to derive the score for emergency risk prediction to estimate the probability of mortality during an inpatient stay [30].

### Objective

By leveraging large-scale electronic health records (EHRs) and machine learning, we intend to create an innovative, dynamic, and interpretable System for Emergency Risk Triage (SERT) for risk stratification in the ED. This protocol describes the detailed data collection procedures, data manipulation, and predictive modelling to accomplish our goals. In particular, we will employ the AutoScore framework to construct a dynamic SERT for risk assessment at multiple decision points in the ED. Our solution will also be compared with traditional clinical triage tools and black box machine learning algorithms.

## Methods

### Study Setting

This is a large-scale, retrospective, single-center study conducted in Singapore. As a city-state in Southeast Asia with an approximately 5.4 million population, Singapore provides affordable health care through partial subsidies and co-payments. The study site, Singapore General Hospital, is Singapore’s largest and oldest tertiary referral hospital, with 1700 inpatient beds and over 30 clinical specialties. Each year, its ED sees more than 120,000 visits and admits 36,000 patients for inpatient care [16,31].

At public hospitals in Singapore, patients visiting EDs are triaged based on their symptoms according to the national PACS [32]. PACS-1 refers to patients who are seriously ill and require immediate medical care, PACS-2 refers to nonambulant patients who do not appear to be at risk of collapse, PACS-3 refers to ambulant patients, and PACS-4 refers to nonemergency cases. An initial triage is often recommended and used to identify patients who are more acutely ill and need immediate attention. As soon as resuscitation is required, the patient is taken directly to the resuscitation area. Otherwise, the patient will be directed either to a critical care area or a waiting area, depending on the patient’s condition.

### Study Cohort and Design

**Figure 1:**
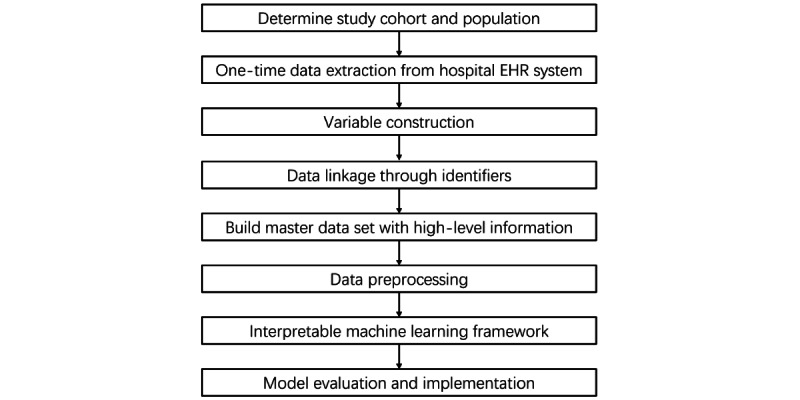
Flowchart of the study design. EHR: electronic health record.

**Figure 2:**
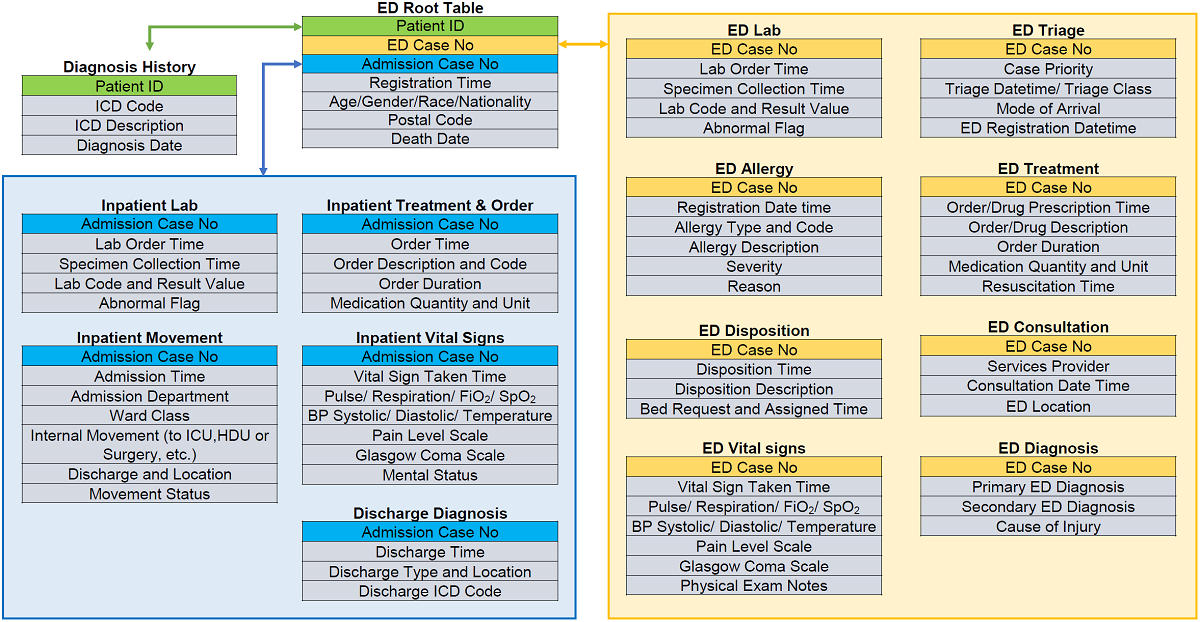
Illustration of the data linkage process of raw data tables through 3 primary identifiers. BP: blood pressure; ID: identification; ICD: International Classification of Diseases; ED: emergency department; ICU: intensive care unit; HDU: high dependency unit; SpO2: peripheral oxygen saturation; FiO2: fraction of inspired oxygen.

The flowchart of the entire project is shown in Figure 1. In the extracted data set, there are 3 primary identifiers: “*ED Case No,” “Admission Case No,”* and “*Patient ID,”* to represent the unique ED visit, the admission case, and the patient, respectively. Figure 2 illustrates how variables are constructed from and linked to these 3 identifiers. By consolidating the selected variables, a master data set will be created. Afterwards, the constructed master data set will be processed with outlier removal and missing value handling. The interpretable machine learning framework will then be implemented, and the models will be evaluated and compared with other baseline approaches, including traditional clinical scores, machine learning, and deep learning.

Singapore Health Services’ Centralized Institutional Review Board approved this study (CIRB Ref: 2021/2122), and a waiver of consent was granted to collect and analyze EHRs.

### Data Source and Extraction

Study subjects have been drawn from the hospital’s EHRs using the SingHealth-IHiS Electronic Health Intelligence System, which combines data from multiple clinical, operational, and finance data sources [33]. Before analysis, all data, including the 3 primary identifiers, have been de-identified to ensure that they are sufficiently anonymous. Records of deaths are obtained from the national death registry and are matched to specific patients in our database. Relevant variables are extracted from the beginning of the ED visits until the end of the patient’s journey. Moreover, patients’ medical histories are extracted and matched for each unique patient through *“Patient ID.”* The extracted data were saved in multiple CSV files for subsequent processing and analysis.

### Data Cleaning and Preprocessing

Data extracted from EHRs may contain many erroneous entries, as the EHRs are designed for clinical use and not explicitly modified for research purposes. This results in a lot of noise, missing values, outliers, and duplicate or incorrect records due to system problems or clerical errors. These issues will be addressed in several ways. First, wholly duplicated entries will be removed. Second, if the vital signs or laboratory test results are outside the normal range, they are considered outliers. All outliers are marked as missing values and are handled by appropriate imputation methods (eg, the mean or median value imputation based on the training data set). Third, a descriptive analysis will be conducted to determine whether the overall percentage and number are within a reasonable range.

### Variable Construction

Candidate variables have been identified based on expert opinions as well as relevant literature [18,30,34-36]. Moreover, we have sought input from clinicians and informaticians familiar with the raw data to determine which features are feasible to extract and construct from the sources. The general rationale is to include all ED-relevant variables of high quality. Therefore, irrelevant, repeated, or largely missing variables will be excluded. For time-series data (such as laboratory test results and vital signs), the first, last, and average measurements are extracted and constructed for each ED episode. Past health care utilization will be derived per the patient’s medical history.

Table 1 presents a list of high-level constructed variables. These variables are classified into 6 main categories depending on the time frame during which the variables could be collected: past medical history, ED triage, ED disposition, within the first 24 hours of inpatient stay, inpatient discharge, and after inpatient discharge. Variables of patient data include demographics, comorbidities, drug history, presenting vital signs, essential laboratory results, and treatments administered in the ED. There are also nonpatient variables such as ED waiting time from triage to consultation, ED boarding time (from consultation to ED disposition), patient load in the ED (number of other patients registered in the ED at that time), time of the day, and day of the week.

### Outcomes

The clinical outcomes in this study include the following adverse events experienced by patients during their inpatient stay:

1. Admission: A hospital admission following an ED visit [37-39]. Each ED attendance is classified as admission or discharge according to the clinical decision made. As a result, patients who left before a decision could be made are excluded rather than considered discharged.
2. Inpatient death: A clinically certified death of a patient admitted to the hospital and who died during the hospitalization.
3. 2/7/30-day mortality: A clinically certified death of an admitted patient that occurred 2/7/30 days after the ED visit regardless of the place of death.
4. ICU transfer: Identified using the hospital’s admission, transfer, and discharge database. Whenever a patient had more than one transfer from ward to ICU, only the data before the first transfer were included.
5. Cardiac arrest: Defined as the loss of a palpable pulse with attempted resuscitation in the ward.
6. Prolonged hospital length of stay: Defined as 21 days or more for the hospital stay.

**Table 1:** List of the high-level constructed variables in the master data set, along with their sources and categories.

Category	Subcategory	Source table	High-level variables extracted
**Patient history**			
	Health care utilization summary	Inpatient movement, ED^a^ root table	Count of ED visits, emergency admissions, surgeries, ICU^b^ or HDU^c^ transfer in the patient’s history (past 30/90/180/365 days)
	Comorbidities	Diagnosis history	Charlson Comorbidity Index (17 variables; chronic disease), Elixhauser Comorbidity Index (30 variables)
**Information collected at triage station**			
	Demographics	ED root table	Age, gender, race, nationality, postal code
	ED-prehospital	ED triage	Mode of arrival, high priority (chest pain/suspected stroke case), fever or not
	ED–triage information	ED triage	Triage waiting time, triage class (Patient Acuity Category Scale system), time of the day (midnight or not), day of the week (weekend or not)
	Triage vital signs	ED vital signs	Pulse, respiration, SpO_2_^d^, systolic BP^e^, diastolic BP, temperature
**Information collected at ED disposition**			
	ED vital signs	ED vital signs	Vital measurement frequency and major ED vital readings: pulse, respiration, fraction of inspired oxygen, SpO_2_, systolic BP, diastolic BP, temperature, pain level scale, Glasgow coma scale, alert (extracted from physical notes)
	ED laboratory	ED laboratory	Laboratory measurement frequency and major laboratory test results: potassium, creatinine, sodium, bicarbonate, albumin, creatine kinase-MB (mass), creatine kinase, prothrombin time, N-terminal pro–B-type natriuretic peptide, C-reactive protein
	ED consultation and treatment	ED consultation, ED treatment	Services provider, consultation waiting time, ED location, length of consultation, resuscitation, major emergency surgeries, pre-selected major ordering
	ED allergy	ED allergy	Major allergy types and reasons, severity
	ED disposition and diagnosis	ED disposition, ED diagnosis	Disposition type, major primary diagnosis, secondary diagnosis (eg, trauma)
	Outcomes	ED disposition, ED root table	Admissions, mortality within ED, direct transfer to ICU
**Information collected within the first 24 hours of inpatient stay**			
	Inpatient stay patient flow	Inpatient movement	ICU or HDU admission, ward class, duration of ICU or HDU stay, hospital departments, surgeries
	Inpatient vital	Inpatient vital signs	Pulse, respiration, SpO_2_, systolic BP, diastolic BP, temperature, Glasgow coma scale, height, weight, BMI
	Inpatient laboratory	Inpatient laboratory	Laboratory measurement frequency and major laboratory test results: albumin, potassium, creatinine, sodium, bicarbonate, creatine kinase, creatine kinase_MB (mass), C-reactive protein, prothrombin time, procalcitonin, blood PH, glycated hemoglobin A1c, triglycerides, cholesterol, high-density lipoprotein cholesterol, low-density lipoprotein cholesterol
	Inpatient treatment	Inpatient treatment and order	Major medication prescription and order
**Information collected at discharge**			
	Health care utilization summary	Inpatient movement	Count of ED visits, ED admissions, surgeries, ICU or HDU admissions last year
	Discharge information	Discharge diagnosis	Primary discharge diagnosis, discharge location, length of stay
	Outcomes	Inpatient movement, discharge diagnosis	ICU transfer, inpatient mortality, cardiac arrest, prolonged hospital length of stay
**Information collected after discharge**			
	Outcomes	ED root table	2/7/30-day mortality, emergency readmission, ED revisit

^a^ED: emergency department.

^b^ICU: intensive care unit.

^c^HDU: high dependency unit.

^d^SpO_2_: peripheral oxygen saturation.

^e^BP: blood pressure.

### Predictive Modelling for Clinical Decision Making

In this study, we will develop and validate a novel interpretable triage system for risk stratification of patients in the ED. Our proposed solution will be compared with baseline risk prediction tools such as traditional clinical scores and black box machine learning models. The extracted data set will be split into training, validation, and testing sets to build and validate the predictive models. The ED visit episodes from January 1, 2008, to December 31, 2018, will be randomly divided into 2 non-overlapping cohorts: a training cohort (80%) and a validation cohort (20%). The ED visits dated in 2019 are assigned to one testing cohort, while those dated in 2020 are assigned to a second testing cohort covering the period of the COVID-19 pandemic [40,41]. Using this sequential testing design, we will be able to test whether the population shift and the COVID-19 pandemic would impact model performance [42]. Further details are presented below.

#### Proposed Method: Interpretable SERT

SERT consists of 3 scoring algorithms, each tailored to its application at different time points in the ED. On arrival at the triage station, SERT-1 is used to estimate patients’ likelihood of admission (inpatient and ICU) and 2-day mortality. SERT-1 is intended to assess the patient’s immediate urgency based on basic patient information, simple vital measurements, and medical histories readily available during triage. While in the ED, SERT-2 predicts patients’ admission (inpatient and ICU) and 2/7-day mortality using a variety of variables, including laboratory test results, vital signs, ED treatment, diagnosis, and some administrative information. As an extension of the SERT-1 algorithm, SERT-2 incorporates additional variables obtained during ED stay to better predict outcomes. On admission, SERT-3 predicts the likelihood of 7/30-day mortality, ICU transfer, and prolonged length of stay using variables collected in the ED and during the first 24 hours of inpatient stay. In actual clinical implementation, in the case where a patient has incomplete information, SERT will use imputation methods to fill in the missing values before calculating the risk score. In summary, SERT allows for a comprehensive risk assessment and prediction in the ED in a dynamic manner.

The clinical risk-scoring models have been traditionally developed in 2 ways: through expert opinions or consensus and conventional cohort studies. However, both approaches are labor-intensive and are not easy to update over time. Recently, we developed an interpretable machine learning–based automatic clinical score generator, AutoScore, as a practical and universal solution for risk scoring [29]. Using the AutoScore framework, users could seamlessly generate parsimonious risk models (ie, point-based sparse risk scores), thereby supporting automated machine learning solutions in health care [43]. AutoScore comprises 6 modules. In module 1, random forest is used to rank variables in terms of their contribution to modelling. Module 2 categorizes continuous variables to address nonlinearity and facilitate the generation of point-based scores. Module 3 computes scores based on a subset of variables and logistic regression, while module 4 determines the optimal number of variables based on a parsimony plot. Module 5 enables fine-tuning of the cut-off values for categorizing continuous variables for preferable interpretation, and module 6 provides a final performance evaluation. AutoScore is used to develop the 3 SERT scoring algorithms with the candidate variables and the outcomes.

#### Baseline Methods: Traditional Clinical Scores

Several traditional clinical scores will be calculated for performance comparison with the SERT scores. They are the PACS triage system [32], Modified Early Warning Score [44], National Early Warning Score [45], Rapid Acute Physiology Score [46], Rapid Emergency Medicine Score [47], and Cardiac Arrest Risk Triage [48].

#### Baseline Methods: Black Box Machine Learning Models

Additionally, several machine learning techniques will be compared as baselines for predictive modelling. Of the many machine learning algorithms, we will apply the following popular ones as examples.

1. Random forest [49]: As the most commonly used tree-based prediction tool, its R package “RandomForest” will be used for model fitting. The parameters will be selected based on recommendations made in previous literature [50,51], where *ntree*= 100 and *mtry* is the principal square root of *m* (*ntree* number of trees grown; *mtry*: number of variables randomly sampled as candidates at each split).
2. Least absolute shrinkage and selection operator [52]: As a penalized regression technique, it is another popular method used in clinical modelling. It is a regression-based method that employs a regularization process for variable selection to increase the statistical model’s predictive accuracy and interpretability. In our study, its regularization rate will be optimized through 10-fold cross-validation.
3. Deep learning [53]: As a branch of the machine learning field that uses deep neural networks, deep learning was initially widely adopted for computer vision and image understanding before being used for medical image analysis. More recently, researchers have begun to explore deep learning for EHR analysis [54,55]. We are particularly interested in applying deep learning algorithms for adverse event prediction, drawing on the rich sources of EHR data, as described earlier. Using the PyTorch library, we will construct a long short-term memory network [56]. In addition, a multilayer perceptron [57] will be used in conjunction with long short-term memory to learn nontemporal data.

### Model Comparison and Performance Metrics

To evaluate the performance of all predictive models, receiver operating characteristic (ROC) analysis will be conducted on the 2 testing cohorts. An overall measure of predictive performance is represented by the area under the ROC curve. Moreover, we will calculate the measures of diagnostic accuracy, such as sensitivity, specificity, positive predictive value, and negative predictive value. These specific measures are determined by setting thresholds on each ROC curve. To achieve optimal balance between sensitivity and specificity, we will select the cut-off points closest to the plot area’s upper-left corner. The 95% CIs for each model or score will also be reported and compared.

### Statistical Analysis

We will perform data analysis using R version 4.0 (R Core Team). When summarizing descriptive results, frequency and percentages are reported for categorical variables, while means and SDs are reported for continuous variables. For categorical variables, the chi-square test or Fisher exact test will be used. For numeric variables, the *t* test will be applied. Further, univariable and multivariable logistic regressions will be used to identify common risk factors associated with the outcomes.

## Results

The raw data have been extracted, and we are currently linking and cleaning the data. In the data extraction process, we included all patients who visited the ED at Singapore General Hospital between January 1, 2008, and December 31, 2020. Patients under the age of 21 years were excluded. If the patients were admitted through the corresponding ED visit, they would be followed throughout their inpatient stay. The data set contains more than 1.8 million ED visit episodes of over 810,000 unique patients. Approximately 650,000 of these ED visits resulted in subsequent hospitalizations. Our findings and modelling results are expected to be published by 2022.

## Discussion

This paper presents a protocol designed to leverage large-scale EHRs and advanced machine learning techniques for risk stratification and triage in the ED. Among numerous ED triage and risk prediction scores and tools, our proposed SERT solution is unique and innovative because of its dynamic nature and modelling transparency. This project will build on the success of our previous research on risk modelling with EHRs for patients in the ED [14,16,30].

### Significance

The identification of patients’ risk at an early stage allows for better resource allocation. There is particular significance in this point because the instability of vital signs may occur later in the ward, leaving a limited time window for life-saving action or decision making, which can be especially difficult in a busy hospital. Patient groups at high risk should be identified earlier in the ED and, if possible, flagged for more stringent monitoring. Similarly, low-risk patients may require less intensive monitoring and treatment, thereby saving hospital resources. The SERT system that we propose has the potential to provide a feasible solution. This system allows medical personnel to assess patient risk at multiple decision points based on various clinical and nonclinical factors. In a dynamic way, SERT measures risk sequentially and in a manner that is perfectly suited to actual clinical needs.

### Strengths

First, this study uses a large set of EHR data over a 13-year period, which contains comprehensive patient information. As Singapore’s largest hospital, Singapore General Hospital provides medical care to a wide range of patients throughout the country; thus, its EHRs ensure good coverage for a large population. Additionally, the longitudinal data allow us to validate the SERT system using data before and after COVID-19. Thus, we will have the opportunity to evaluate the impact of the global pandemic on triage performance in the ED. The insights gained from system evaluation could be used to examine possible model adaptations in shifted clinical settings.

Second, the SERT triaging system we intend to develop will be transparent and easily understandable. All 3 SERT scores are parsimonious and point-based, as only the most significant variables are considered in their formulation. Their formats follow the same convention as widely used clinical scores such as the National Early Warning Score and Modified Early Warning Score, allowing for easy comprehension and quick adoption. In contrast, black box machine learning models are challenging to comprehend, making them inaccessible to clinicians [25]. Although there are techniques for post hoc model explanation, most machine learning models are not inherently interpretable [25].

Third, this project aims to develop a dynamic system capable of identifying risk strata at different decision points in the ED. During the initial triage process and the patient’s stay in the ED, SERT predicts the likelihood of inpatient and ICU admissions. Whenever variables are altered, the scores can be updated, making the risk assessment dynamic and practical. In addition, SERT can make mortality predictions to assess the likelihood of the worst outcomes for patients who will be hospitalized.

Lastly, the simple form of the scores in SERT permits a variety of implementation schemes. As an example, the actual implementation can be as simple as a mobile app. Users may input relevant information into the app, which will return a risk score at the time of inquiry. The SERT scoring platform can also be easily integrated into existing information technology systems, which requires only simple calculations and therefore little computing power. The application can be designed and implemented in real time, similar to that seen in a recent study in the United States [13].

### Limitations and Future Plan

Although the study site is the largest hospital in the country, the SERT system may not apply to international institutions where EDs operate differently. We intend to conduct cross-institutional validation of our system with both local and international partners. In the case that our SERT system is not feasible, the methods we use can easily be adapted to any context because AutoScore is a generic, universal scoring tool that permits the creation of interpretable clinical scores. In addition, we anticipate a sparse data set with numerous missing values, particularly for comorbidities, medications, and time series records of vitals and laboratory test results. To address the issue of data sparsity, we will examine various data imputation strategies and feature representation techniques.

Our future efforts will include identifying opportunities to conduct a rigorously designed randomized trial to evaluate the system. In the long-term, we hope to expand the evaluation to a multicenter trial involving several countries.

### Conclusions

Clinical decision making has widely benefited from the use of machine learning techniques. However, the black box models created by these methods prevent their use in actual clinical practice. Our study aims to address this issue by proposing an innovative SERT scoring system. An interpretable machine learning–based AutoScore framework will be used to create a series of 3 SERT scores that can be used in the medical setting at various decision points throughout the patient’s journey. The SERT system is notable for its dynamic nature and transparency. If validated successfully, it will establish a standard for data processing and modelling by utilizing large-scale EHRs and interpretable machine learning. The proposed system may be well suited to bridge the gap between advanced computation and clinical applications.

## Abbreviations

AI: artificial intelligence
ED: emergency department
EHR: electronic health record
ICU: intensive care unit
PACS: Patient Acuity Category Scale
ROC: receiver operating characteristic
SERT: System for Emergency Risk Triage
